# Propofol inhibited autophagy through Ca^2+^/CaMKKβ/AMPK/mTOR pathway in OGD/R-induced neuron injury

**DOI:** 10.1186/s10020-018-0054-1

**Published:** 2018-11-23

**Authors:** Bei Sun, Hao Ou, Fei Ren, Ye Huan, Tao Zhong, Min Gao, Hongwei Cai

**Affiliations:** 1 Department of Anesthesiology, Xiangya Hospital, Central South University, 410078 Changsha, Hunan People’s Republic of China; 2 Department of Emergency and Critical Care Medicine, The Third Xiangya Hospital, Central South University, 410013 Changsha, Hunan People’s Republic of China; 3 Translational Medicine Center of Sepsis, Department of Pathophysiology, The Third Xiangya Hospital, Central South University, 410013 Changsha, Hunan People’s Republic of China

**Keywords:** Propofol, Oxygen-glucose deprivation and re-oxygenation, Autophagy, Ca2+/CaMKKβ/AMPK/mTOR

## Abstract

**Background:**

The neuroprotective role of propofol (PPF) in cerebral ischemia-reperfusion (I/R) has recently been highlighted. This study aimed to explore whether the neuroprotective mechanisms of PPF were linked to its regulation of Ca^2+^/CaMKKβ (calmodulin-dependent protein kinase kinase β)/AMPK (AMP-activated protein kinase)/mTOR (mammalian target of rapamycin)/autophagy pathway.

**Methods:**

Cultured primary rat cerebral cortical neurons were treated with oxygen-glucose deprivation and re-oxygenation (OGD/R) to mimic cerebral I/R injury in vitro.

**Results:**

Compared with the control neurons, OGD/R exposure successfully induced neuronal I/R injury. Furthermore, OGD/R exposure notably caused autophagy induction, reflected by augmented LC3-II/LC3-I ratio and Beclin 1 expression, decreased p62 expression, and increased LC3 puncta formation. Moreover, OGD/R exposure induced elevation of intracellular Ca^2+^ concentration ([Ca^2+^]i). However, PPF treatment significantly antagonized OGD/R-triggered cell injury, autophagy induction, and [Ca^2+^]i elevation. Further investigation revealed that both autophagy induction by rapamycin and [Ca^2+^]i elevation by the Ca^2+^ ionophore ionomycin significantly reversed the PPF-mediated amelioration of OGD/R-triggered cell injury. Importantly, ionomycin also significantly abrogated the PPF-mediated suppression of autophagy and CaMKKβ/AMPK/mTOR signaling in OGD/R-exposed neurons. Additionally, activation of CaMKKβ/AMPK/mTOR signaling abrogated the PPF-mediated autophagy suppression.

**Conclusion:**

Our findings demonstrate that PPF antagonized OGD/R-triggered neuronal injury, which might be mediated, at least in part, via inhibition of autophagy through Ca^2+^/CaMKKβ/AMPK/mTOR pathway.

**Electronic supplementary material:**

The online version of this article (10.1186/s10020-018-0054-1) contains supplementary material, which is available to authorized users.

## Background

Cerebral ischemia-reperfusion (I/R) injury is characterized by an insufficient oxygen supply and restoration of blood flow and serves as the main cause for the aggravation of cerebral injury and functional impairment (He et al. 2016). This injury involves complex and multi-factorial mechanisms, including intracellular Ca^2+^ overload and damage to oxidative stress (Chen et al. 2017; Liu et al. 2011). Interestingly, it has been shown that several anesthetic drugs, including isoflurane and propofol (PPF), exert neuroprotective influence on cerebral I/R injury (Li et al. 2013; Wang et al. 2016).

PPF (2, 6-diisopropylphenol) is an intravenous sedative-hypnotic agent and has been extensively utilized in general anesthesia and sedation (Wahr et al. 2000). In addition to its sedative and hypnotic effects, the neuroprotective role of PPF in transient global and focal cerebral I/R has recently been highlighted in both animal and cell models (Wang et al. 2016; Wang et al. 2011). The mechanisms responsible for the neuroprotection of PPF involve the inhibition of apoptosis (Tao et al. 2016), Ca^2+^ overload (Wang et al. 2016), oxidative stress injury (Yu et al. 2018), and so forth.

Recent studies concerning the neuroprotective mechanisms of PPF have focused on autophagy (Cui et al. 2012; Noh et al. 2010). Autophagy is a basic catabolic mechanism which degrades intracellular components and damaged organelles to maintain intracellular homeostasis (Choi et al. 2013). At present, the exact role of autophagy in ischemic brain injury remains controversial (Peng et al. 2018; Hua et al. 2018; Zheng et al. 2018). It has been demonstrated that increased autophagy acts as a compensatory protective mechanism for I/R injury (Su et al. 2014). In contrast, another study has suggested that inhibition of autophagy reduces infarct volume, brain edema, and motor deficits, in a rat model of permanent focal cerebral ischemia (Wen et al. 2008). Of note, PPF has been shown to prevent cerebral ischemia-triggered autophagy activation and cell death in the rat hippocampus after a cerebral I/R insult (Cui et al. 2013). Recent studies have emphasized the correlation between autophagy and Ca^2+^. It has been demonstrated that accumulation of intracellular Ca^2+^ concentration ([Ca^2+^]i) activates CaMKKβ (calmodulin-dependent protein kinase kinase β)/AMPK (AMP-activated protein kinase) signaling cascade, leading to mTOR (mammalian target of rapamycin) signaling inhibition, and thus autophagy induction (Shi et al. 2018; Zhang et al. 2015; Lee et al. 2012).

Based on the aforementioned studies, we hypothesized that PPF might play a neuroprotective role in cerebral I/R injury through inactivation of Ca^2+^/CaMKKβ/AMPK/mTOR/autophagy pathway. To test this hypothesis, cultured primary rat cerebral cortical neurons were treated with oxygen-glucose deprivation and re-oxygenation (OGD/R) to mimic cerebral I/R injury in vitro. We first explored the effects of PPF on OGD/R-induced neuronal injury in rat primary cerebral cortical neurons. Afterwards, we verified whether the neuroprotective mechanisms of PPF were associated with the regulation of Ca^2+^/CaMKKβ/AMPK/mTOR/autophagy pathway.

## Methods

### Ethics statement

All animal-related experiments in this study were approved by the Ethics Committee for Animal Experimentation of Xiangya Hospital, Central South University and were performed in accordance with the guidelines for the Care and Use of Laboratory Animals of the National Institutes of Health.

### Primary culture of cerebral cortical neurons

Primary rat cerebral cortical neurons were isolated from the cerebral cortex of fetal Sprague-Dawley rats at embryonic day 17 as previously described (Liu et al. 2018), with some alterations. Briefly, the dissected cortices were dissociated with 0.125% trypsin (Gibco, Grand Island, NY, USA) under sterile conditions at 37 °C for 10 min. Neurons were suspended in Dulbecco’s Modified Eagle’s Medium (DMEM, Gibco, Thermo Fisher Scientific, Inc., Waltham, MA, USA) containing 10% fetal bovine serum (FBS, Gibco) to inactivate the trypsin, and then filtered through a cell strainer. Afterwards, cells were seeded into 6-well plates or coverslips those were pre-coated with poly-D-lysine (Sigma-Aldrich, St. Louis, MO, USA). After 24 h of incubation, the medium was replaced with Ca^2+^-free and serum-free Neurobasal medium supplemented with 2% B27 and 0.5 mM glutamine (Gibco), and half of the medium was changed every 2–3 days. Cells were maintained in humidified air with 5% CO_2_ at 37 °C. All experiments were performed on neurons after 10 days in culture. To determine the purity of neurons, cerebral cortical neurons were subjected to neuron-specific enolase (NSE) staining under fluorescent microscopy (Leica, DMI 4000 B, Japan) at day 10 after the culture.

### Neuronal I/R injury model induced by OGD/R and drug treatment

To mimic cerebral I/R in vitro, oxygen-glucose deprivation/reperfusion (OGD/R) was performed as described previously (Liu et al. 2018). Oxygen-glucose deprivation (OGD): Primary cerebral cortical neurons were washed twice with phosphate-buffered saline (PBS, pH 7.4) and then refreshed with the glucose-free DMEM (Gibco). After that, cell cultures were transferred into a hypoxia chamber (containing a gas mixture of 5% CO_2_ and 95% N_2_) at 37 °C to produce OGD condition in cultured neurons.

Reperfusion (R): After 4 h of incubation in the hypoxic chamber (the above-mentioned OGD process), the neurons were incubated again in Neurobasal medium containing 2% B27 and 0.5 mM glutamine and then returned to the normoxic incubator (95% air/5% CO_2_) for 24 h at 37 °C. Cells without any treatment served as the control group.

For PPF treatment, cultured cortical neurons (5 × 10^5^) were treated with different doses of PPF (0, 0.01, 0.03 and 0.05 mM in 0.1% DMSO; Sigma-Aldrich, St. Louis, MO, USA) during the period of OGD/R.

### Cell viability and LDH secretion

Cell viability and LDH leakage assays were performed to evaluate neuronal damage as previously described (Liu et al. 2018). After OGD/R treatment, primary cultured cortical neurons were incubated with Cell Counting Kit-8 (CCK-8; Sigma-Aldrich) solution for another 4 h. Absorbance was measured using a microplate reader at 450 nm to assess cell viability. In addition, an LDH-Cytotoxicity Assay Kit (Sigma-Aldrich) was used to assess the activity of LDH. Briefly, the culture media of cortical neurons were collected after exposure to OGD/R and then centrifuged to obtain the supernatant. The cells were lysed with 1% Triton X-100 (Sigma-Aldrich) and centrifuged to remove cellular debris. After that, the culture supernatant and cell lysates were respectively incubated with LDH reaction mixture at 37 °C for 15 min. The absorbance was read at 490 nm when the reaction was stopped, and LDH release was expressed as a percentage of total LDH.

### Cell apoptosis assay

An annexin V-fluorescein isothiocyanate (FITC)/propidium iodide (PI) cell apoptosis kit was used according to the instructions (Invitrogen, Thermo Fisher Scientifc, Inc., Waltham, MA, USA) to quantify cell apoptosis. After exposure to OGD/R, the primary rat cortical neurons were collected by trypsinization and washed twice with cold phosphate buffered saline (PBS). After centrifugation at 1,500 rpm for 5 min, the cells were resuspended in binding buffer and then incubated with FITC-labeled Annexin V and PI in the dark for 15 min. Cells were analyzed using the FACScan flow cytometry (BD Biosciences).

### Western blot

Western blot was performed to detect protein expression as previously described (Yuan et al. 2012). Briefly, total proteins from primary rat cortical neurons were isolated using the protein buffer (Beyotime, Shanghai, China). Proteins were then separated by 10% SDS-PAGE and transferred onto PVDF membranes. After being blocked with 5% fat-free milk, the membranes were incubated at 4 °C overnight with primary antibodies against p62 (1:1,000, Abcam, Cambridge, MA, USA), Beclin-1 (1:1,000, Abcam), microtubule-associated protein 1 light chain 3 (LC3-I and LC3-II, both from Anti-LC3B antibodies, 1:1,000, Sigma-Aldrich), CaMKKβ (1:1,000, Abcam), AMPK (1:1,000, Cell Signaling Technology Inc., Danvers, MA, USA), phospho (p)-AMPK (1:1,000, Cell Signaling Technology Inc.), mTOR (1:1,000, Cell Signaling Technology Inc.), and p-mTOR (1:1,000, Cell Signaling Technology Inc.). Subsequently, membranes were incubated for 2 h at room temperature with horseradish peroxidase (HRP)-secondary antibodies in TBST. The protein was visualized using the ECL detection system (Beyotime, Shanghai, China). The band intensity was quantified with Image-Pro Plus 6.0 software.

### Green fluorescent protein (GFP)-LC3 immunofluorescence

Transfection of GFP-LC3 was performed according to the manufacturer’s instructions (Genomeditech, Shanghai, China). Culture medium was removed, washed three times with PBS, and then fixed with 5% paraformaldehyde for 10 min at room temperature. Coverslips were again washed three times with PBS, permeabilized with 0.3% Triton-X for 5 min, and stained with DAPI for 3 min. Finally, the coverslips were washed three times with PBS, once with ddH_2_O, and mounted on tissue slides with mounting medium (Thermo Fisher Scientific, Inc.). Fluorescence images were captured and analyzed under a fluorescence microscope (Nikon Corporation, Tokyo, Japan).

### Intracellular Ca^2+^ concentration assessment

The intracellular Ca^2+^ concentration ([Ca^2+^]i) was assessed using fluorescent dye Fluo-3 AM/Pluronic F127 as previously described (Wang et al. 2016). After the indicated treatment, primary rat cortical neurons were seeded on a cover glass and incubated with 5 μM Fura-3/AM and 0.05% Pluronic F-127 (Invitrogen) for 45 min at 37 °C. The coverslips were then equilibrated in Hanks solution. After incubation at 37 °C for 30 min, the samples were determined by a laser scanning confocal microscope under the excitation 488 nm and emission 525 nm to detect fluorescence intensity.

### *CaMKKβ o*verexpression

The CaMKKβ cDNA fragment was cloned into pcDNA 3.1, generating pcDNA3.1- CaMKKβ. Cultured primary rat cortical neurons were transfected with pcDNA3.1- CaMKKβ using Lipofectamine 2000 (Invitrogen, USA) according to the manufacturer’s instructions.

### Statistical analysis

All statistical analyses were performed using SPSS statistical software package standard version 16.0 (SPSS, Inc., Chicago, IL, USA). The data are presented as the mean ± standard deviation (SD) from three independent experiments. The unpaired Student’s *t*-test was used to analyze differences between two groups. One-way analysis of variance (ANOVA) followed by Tukey’s post hoc test was used to analyze differences among multiple groups. *p* < 0.05 was considered to indicate a statistically significant difference.

## Results

### Neuronal I/R injury model was successfully induced by OGD/R

**Fig. 1:**
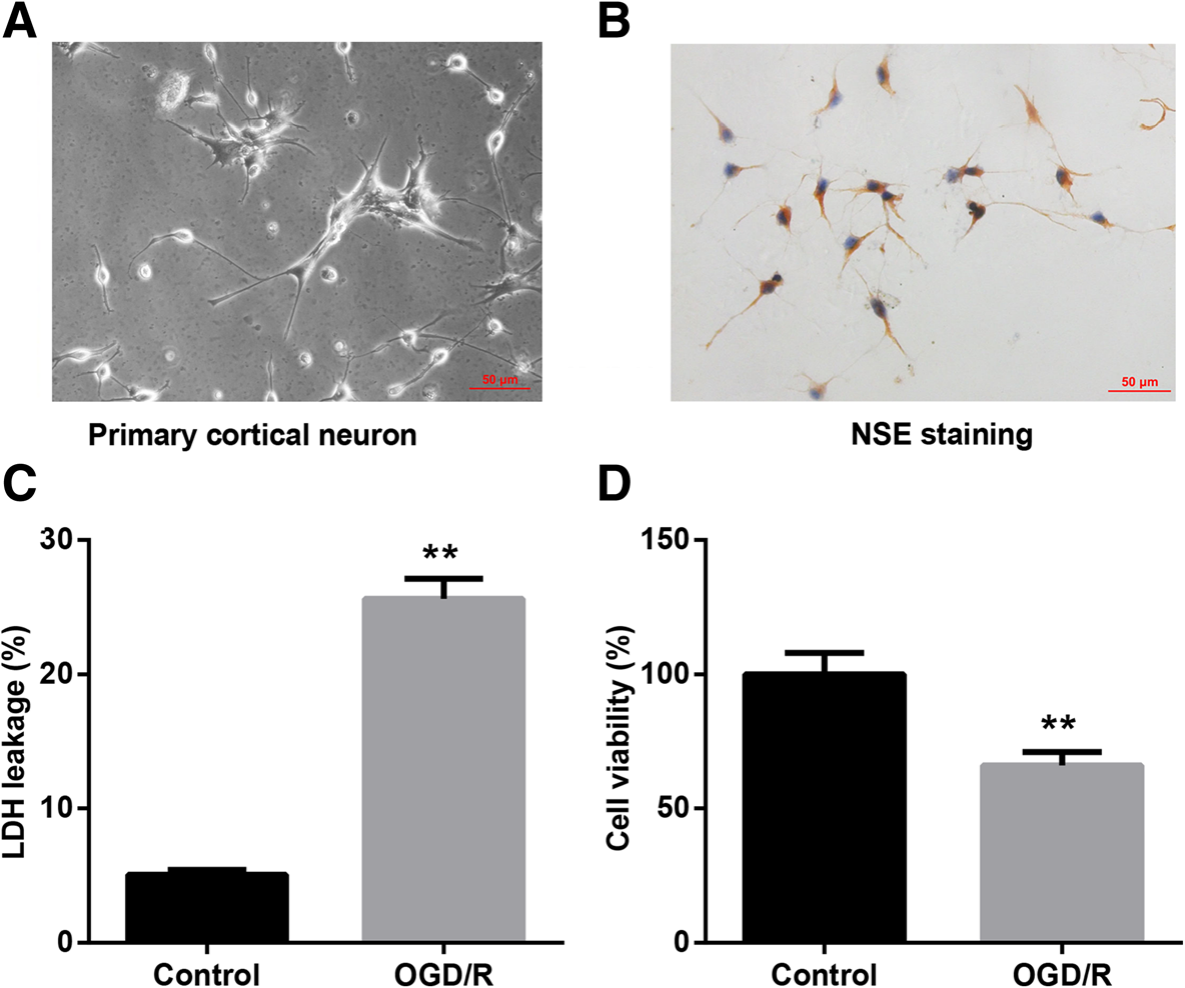
Establishment of neuronal ischemia-reperfusion injury model. Primary cerebral cortical neurons were prepared from Sprague-Dawley rats, and an in vitro cellular model of cerebral ischemia and reperfusion injury was induced by oxygen-glucose deprivation/reoxygenation (OGD/R). **a** Morphological changes of the rat primary cortical neurons cultured for 10 days were observed under a microscope (scale bar: 50 μm). **b** Neuron-specific enolase (NSE) immunohistochemistry staining in cortical neurons (scale bar: 50 μm). **c** Cell injury of cortical neurons exposed to OGD/R was evaluated by lactate dehydrogenase (LDH) release rate, and **d** cell viability was assessed by the CCK-8 assay. Values are represented as the mean ± SD from three independent experiments. ^**^p < 0.01 vs. Control group

After being cultured for 10 days, rat primary cortical neurons exhibited plump cell body, apparent halo, and complete neural network (Fig. 1a), with a high purity indicated by NSE immunohistochemistry staining (Fig. 1b). The cultured primary cerebral cortical neurons were then subjected to oxygen-glucose deprivation for 4 h followed by 24 h of reoxygenation (OGD/R) to mimic cerebral I/R injury in vitro. Afterwards, cell viability and LDH secretion were performed to evaluate the neuronal injury. Data revealed that OGD/R-exposed cortical neurons exhibited notably increased LDH leakage rate and decreased cell viability, as compared with the control cells without OGD/R exposure (Fig. 1c and d). Collectively, these results indicated that OGD/R exposure successfully induced a model of neuronal I/R injury in cerebral cortical neurons.

### PPF antagonized OGD/R-triggered cell injury, autophagy induction and [Ca^2+^]i elevation

**Fig. 2:**
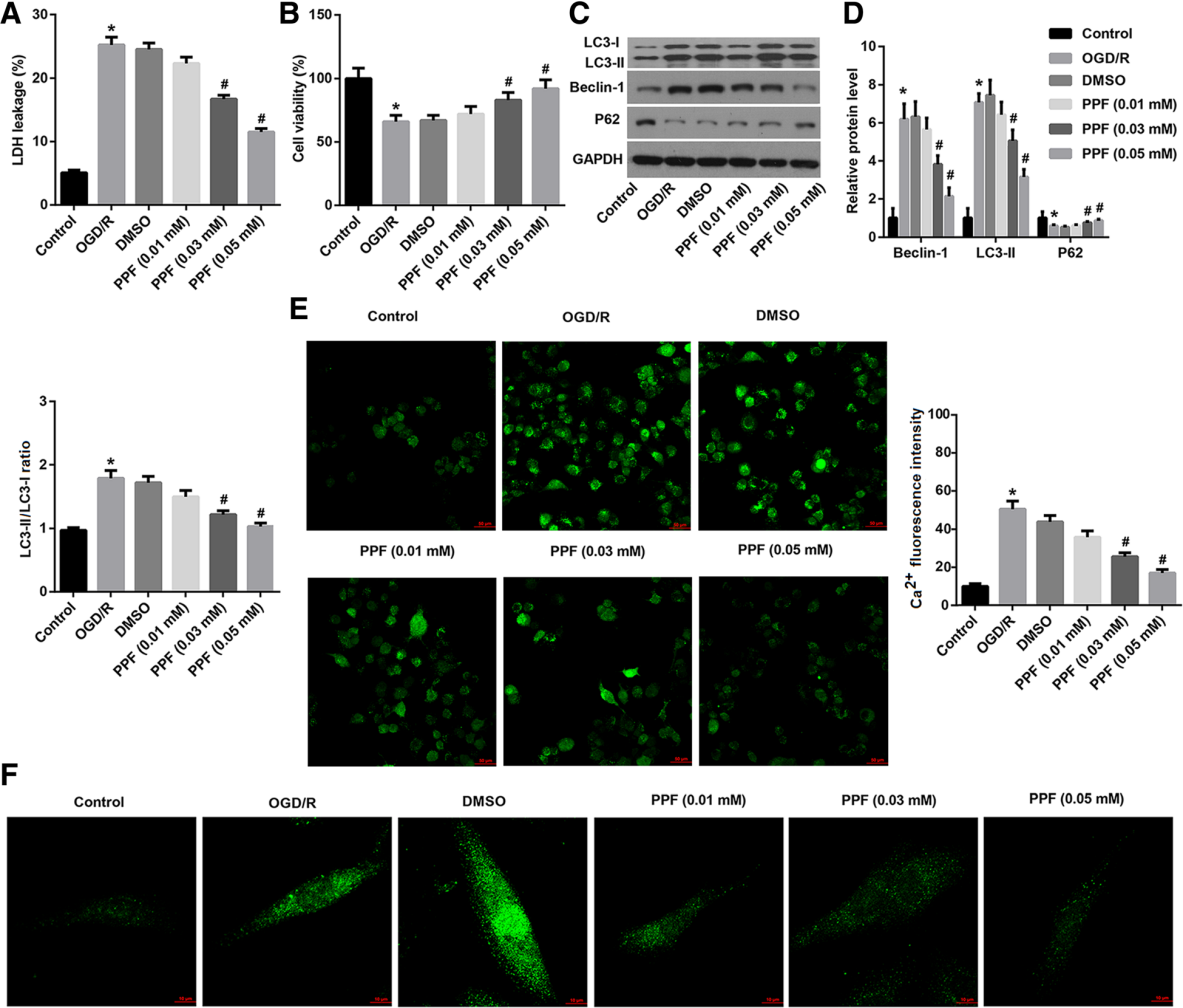
PPF antagonized OGD/R-triggered cell injury, autophagy induction and [Ca^2+^]i elevation. Propofol (PPF) with various concentrations (0.01 mM, 0.03 mM, 0.05 mM in 0.1% DMSO) was added into OGD/R-exposed neurons. **a** LDH release. **b** Cell viability using MTT assay. **c**, **d** Western blot analysis of protein expression of autophagy-related genes LC3, Beclin1, and p62. Quantification of western blots was shown. **e** Ca^2+^ fluorescence images. Scale bar: 50 μm. The qualification of Ca^2+^- fluorescence intensity was shown. **f** The autophagosome puncta of GFP-LC3 by immunofluorescence. Scale bar: 10 μm. Values are represented as the mean ± SD from three independent experiments. ^*^p < 0.05 vs. Control group; ^#^*p* < 0.05 vs. DMSO group

PPF (0.03 and 0.05 mM) significantly antagonized OGD/R-triggered cell injury indicated by inhibition of OGD/R-induced LDH release and restoration of OGD/R-suppressed cell viability by PPF treatment, in a dose-dependent manner (Fig. 2a and b). Furthermore, as shown in Fig. 2c and d, OGD/R exposure greatly induced an increase in protein expression of Beclin-1, LC3-II but induced a decrease in p62. Moreover, the LC3-II/LC3-I ratio, which is indicative of the status of autophagy, was also upregulated by OGD/R exposure. In addition, immunofluorescence images showed strong GFP-LC3 puncta and increased the percentage of GFP-LC3 cells in cortical neurons under OGD/R exposure compared with the control cells (Fig. 2f). These results suggested that OGD/R exposure induced autophagy. However, PPF dose-dependently diminished the OGD/R-induced autophagy level (Fig. 2c, d and f). Moreover, PPF notably reduced the OGD/R-induced intracellular Ca^2+^ concentration ([Ca^2+^]i), as manifested by decreased fluorescence intensity of Ca^2+^ dyed with the explorer Fluo-3, AM and Pluronic F-127 (Fig. 2e).

### Autophagy induction abolished the PPF-mediated amelioration of OGD/R-triggered cell injury

**Fig. 3:**
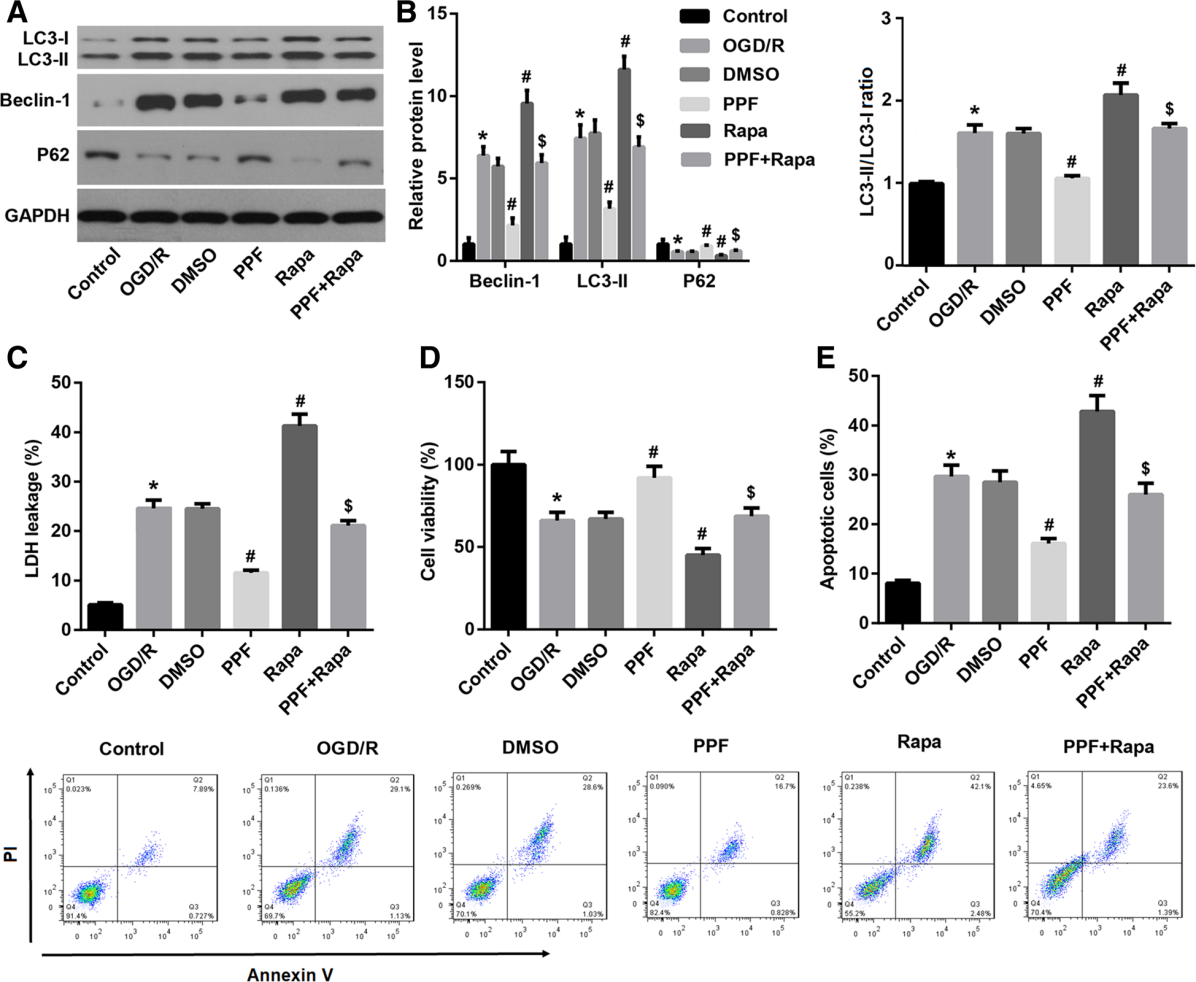
Autophagy induction abolished the PPF-mediated amelioration of OGD/R-triggered cell injury. OGD/R-exposed neurons were pre-treated with the autophagy activator rapamycin (Rapa, 30 μM), followed by propofol (PPF, 30 μM in 0.1% DMSO) treatment. **a**, **b** Western blot was performed to evaluate the effects of autophagy activator rapamycin on protein expression of autophagy-related genes LC3, Beclin1, and p62. Quantification of western blots was shown. **c** LDH release, **d** CCK-8 cell viability, and **e** cell apoptosis assay were performed to evaluate whether autophagy was involved in the PPF-mediated amelioration of OGD/R-triggered cell injury. Values are represented as the mean ± SD from three independent experiments. ^*^p < 0.05 vs. Control group; ^#^*p* < 0.05 vs. DMSO group; ^$^*p* < 0.05 vs. PPF group

To address the role of autophagy in the PPF-mediated amelioration of OGD/R-triggered neuronal injury, we pre-treated OGD/R-exposed neurons with autophagy activator rapamycin, followed by PPF treatment (30 μM in 0.1% DMSO). As indicated in Fig. 3a and b, rapamycin significantly restored the PPF-suppressed autophagy level in OGD/R-exposed neurons, as manifested by an increase in LC3II and Beclin-1 expression, LC3II/LC-I ratio and a decrease in p62 expression. Importantly, activation of autophagy by rapamycin significantly reversed the PPF-mediated inhibition of LDH leakage and cell apoptosis, plus induction of cell viability in OGD/R-exposed neurons (Fig. 3c-e), indicating the involvement of autophagy in the PPF-mediated amelioration of OGD/R-triggered cell injury.

### [Ca^2+^]i abrogated the PPF-mediated amelioration of OGD/R-triggered cell injury

**Fig. 4:**
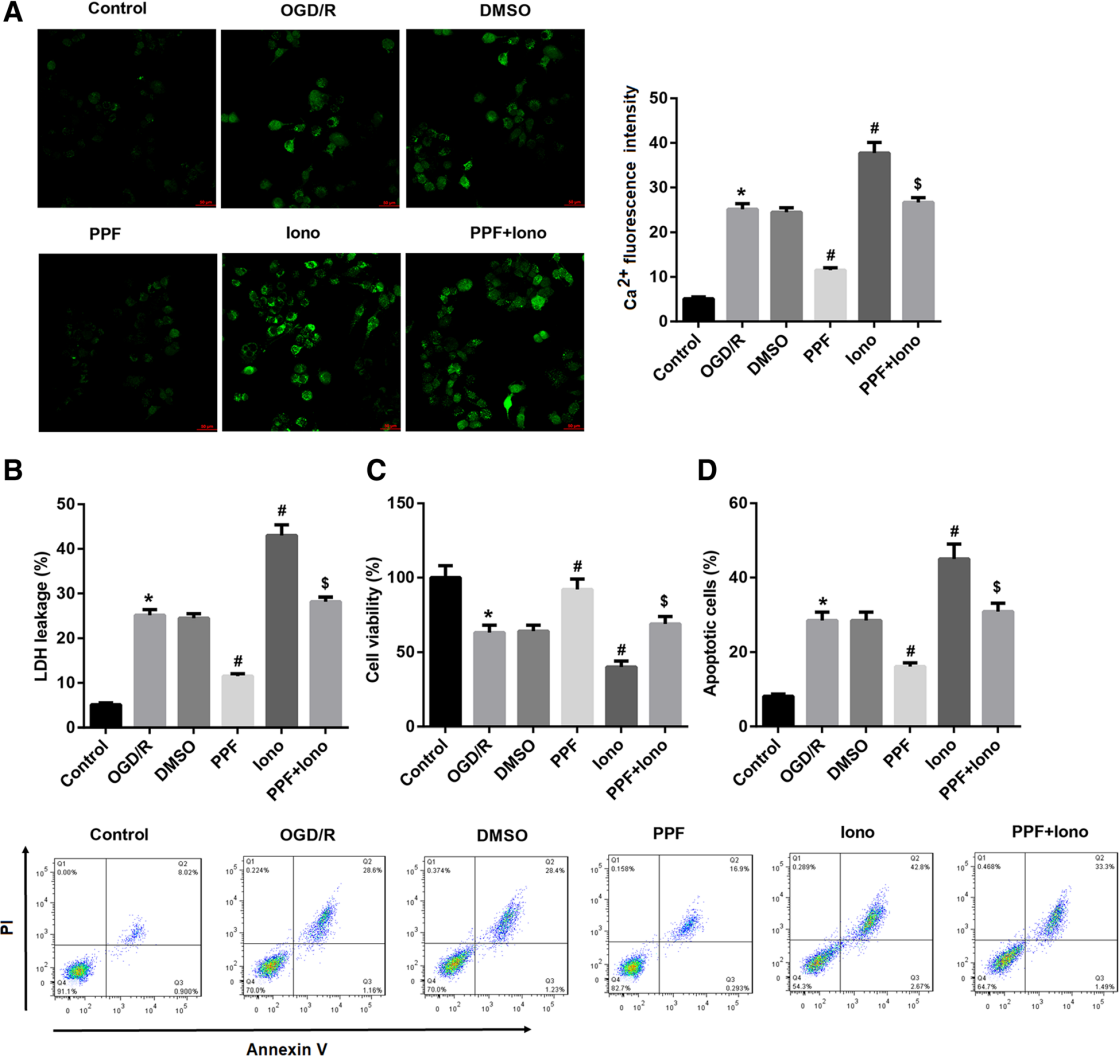
[Ca^2+^]i elevation abrogated the PPF-mediated amelioration of OGD/R-triggered cell injury. OGD/R-exposed neurons were pre-treated with a Ca^2+^ ionophore ionomycin (iono, 0.3 μM), followed by propofol (PPF, 30 μM in 0.1% DMSO) treatment. **a** Ca^2+^ fluorescence images. Scale bar: 50 μm. The qualification of Ca^2+^- fluorescence intensity was shown. **b** LDH release, **c** CCK-8 cell viability and **d** cell apoptosis assay were performed to evaluate whether [Ca^2+^]i was involved in the PPF-mediated amelioration of OGD/R-triggered cell injury. Values are represented as the mean ± SD from three independent experiments. ^*^p < 0.05 vs. Control group; ^#^*p* < 0.05 vs. DMSO group; ^$^*p* < 0.05 vs. PPF group

To determine the role of [Ca^2+^]i in the PPF-mediated amelioration of OGD/R-triggered neuronal injury, we pre-treated OGD/R -exposed neurons with a Ca^2+^ ionophore (ionomycin, 0.3 μM) or an intracellular calcium chelator (BAPTA, 5 μM), followed by PPF treatment (30 μM in 0.1% DMSO). Ionomycin is a calcium ionophore that dose-dependently increases intracellular calcium (Natoli et al. 2014). As expected, ionomycin pre-treatment significantly restored the PPF-suppressed [Ca^2+^]i in OGD/R-exposed neurons, as evidenced by an increase in Ca^2+^ fluorescence intensity (Fig. 4a). Importantly, ionomycin, similar to the autophagy activator rapamycin, significantly reversed the PPF-mediated inhibition of LDH leakage and cell apoptosis, plus induction of cell viability in OGD/R-exposed neurons (Fig. 4b-d). On the other hand, pre-treatment with BAPTA alone, which has been generally used to block intracellular calcium, significantly alleviated OGD/R-triggered cell injury, as indicated by reduced LDH leakage (Additional file 1: Figure S1A) and induced cell viability (Additional file 1: Figure S1B) by BAPTA when compared with the vehicle DMSO group. Furthermore, BAPTA pre-treatment enhanced the PPF-mediated amelioration of OGD/R-triggered cell injury, indicating the [Ca^2+^]i suppression in attenuating OGD/R-triggered cell injury (Additional file 1: Figure S1). Together, these results suggested that [Ca^2+^]i elevation abrogated the PPF-mediated amelioration of OGD/R-triggered cell injury.

### [Ca^2+^]i elevation abolished the PPF-mediated suppression of autophagy in OGD/R-exposed neurons

**Fig. 5:**
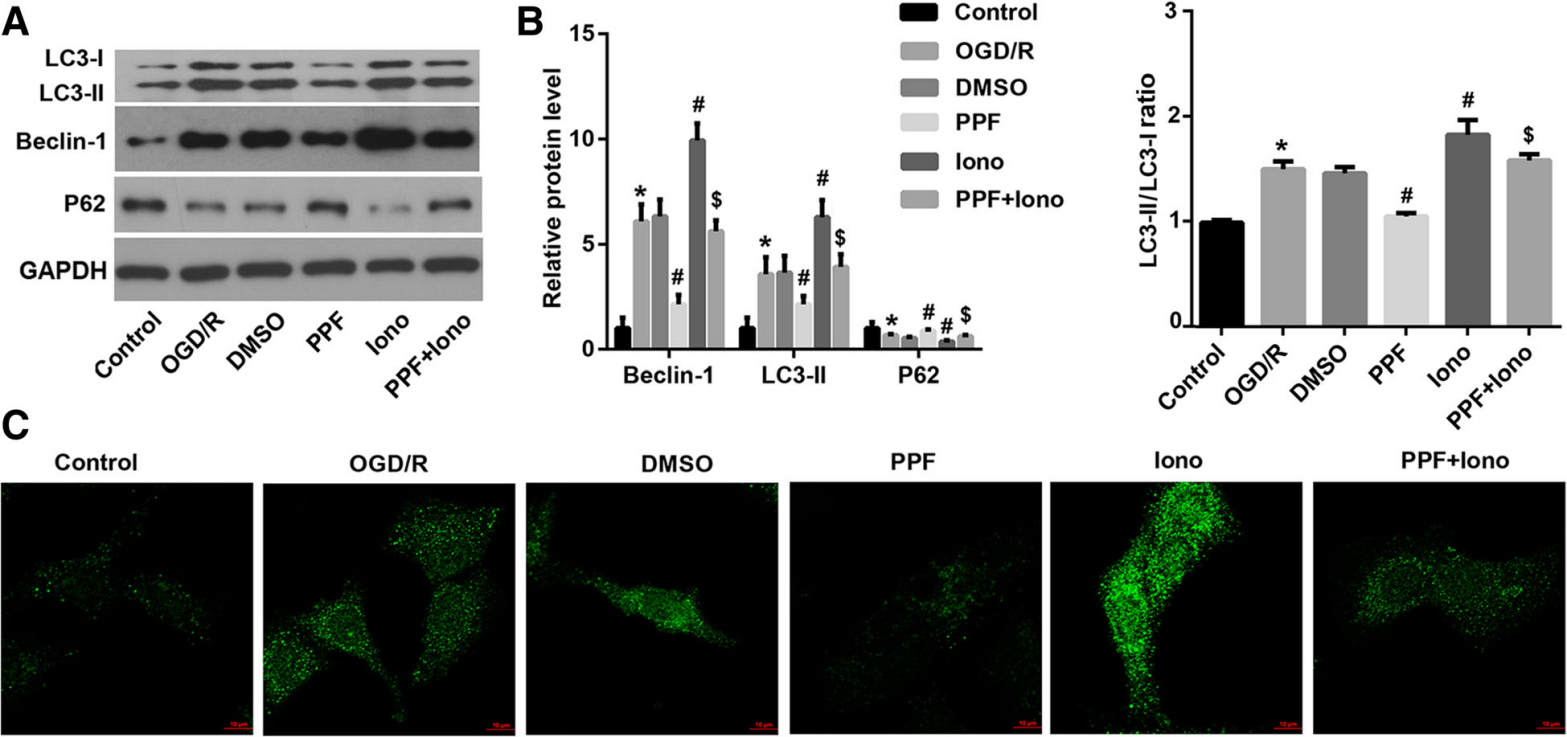
[Ca^2+^]i elevation abolished the PPF-mediated suppression of autophagy in OGD/R-exposed neurons. OGD/R-exposed neurons were pre-treated with a Ca^2+^ ionophore ionomycin (iono, 0.3 μM), followed by propofol (PPF, 30 μM in 0.1% DMSO) treatment. **a**, **b** Western blot was performed to evaluate the effects of [Ca^2+^]i elevator ionomycin on protein expression of autophagy-related genes LC3, Beclin1, and p62. Quantification of western blots was shown. **c** The autophagosome puncta of GFP-LC3 by immunofluorescence. Scale bar: 10 μm. Values are represented as the mean ± SD from three independent experiments. ^*^p < 0.05 vs. Control group; ^#^*p* < 0.05 vs. DMSO group; ^$^*p* < 0.05 vs. PPF group

We then investigated the role of [Ca^2+^]i in the PPF-mediated suppression of autophagy in OGD/R-exposed neurons. The results demonstrated that elevation of [Ca^2+^]i by ionomycin significantly abrogated the PPF-mediated suppression of autophagy in OGD/R-exposed neurons, as indicated by an increase in LC3II and Beclin-1 protein expression (Fig. 5a and b) and GFP-LC3 puncta (Fig. 5c), plus a decrease in p62 (Fig. 5a and b) compared with the PPF alone-treated group.

### Ca^2+^/CaMKKβ/AMPK/mTOR signaling was involved in the PPF-mediated suppression of autophagy

**Fig. 6:**
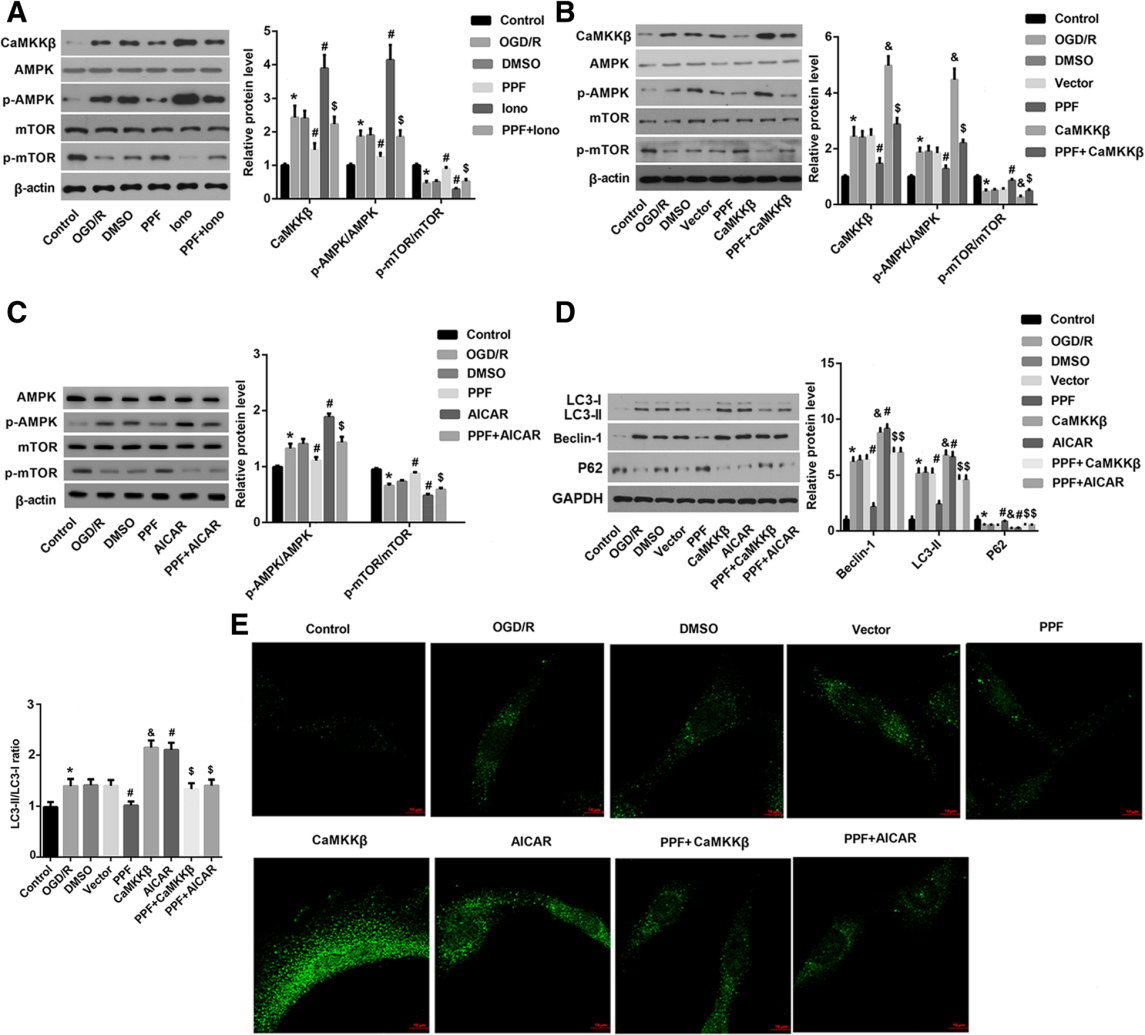
Ca^2+^/CaMKKβ/AMPK/mTOR signaling was involved in the PPF-mediated suppression of autophagy. **a** Western blot was performed to evaluate the effects of [Ca^2+^]i elevator ionomycin (iono) on the PPF-mediated regulation of CaMKKβ, AMPK, p-AMPK, mTOR, and p-mTOR. **b** Western blot was performed to investigate the effects of overexpression of CaMKKβ on the PPF-mediated regulation of CaMKKβ, AMPK, p-AMPK, mTOR, and p-mTOR. **c** Western blot was performed to investigate the effects of activation of the AMPK pathway by AICAR on the PPF-mediated regulation of CaMKKβ, AMPK, p-AMPK, mTOR, and p-mTOR. **d** Western blot was performed to investigate the effects of activation of CaMKKβ/AMPK/mTOR pathway on the PPF-mediated suppression of autophagy-related proteins. **e** GFP-LC3 immunofluorescence was performed to evaluate the effects of activation of activation of CaMKKβ/AMPK/mTOR pathway on the PPF-mediated suppression of autophagy level. Scale bar: 10 μm. Values are represented as the mean ± SD from three independent experiments. ^*^p < 0.05 vs. Control group; ^#^*p* < 0.05 vs. DMSO group; ^&^*p* < 0.05 vs. vector group; ^$^*p* < 0.05 vs. PPF group

Finally, we explored the potential mechanism by which PPT suppressed autophagy. It has been reported that CaMKKβ activates AMPK by phosphorylation in response to increased [Ca^2+^]i, and that AMPK activates autophagy by down-regulating the activity of mTOR (Shi et al. 2018; Zhang et al. 2015; Lee et al. 2012; Zhang et al. 2017). As indicated in Fig. 6a, PPF significantly decreased the OGD/R-induced increase in protein expression of CaMKKβ and phosphorylation level of AMPK. In contrast, PPF obviously increased the OGD/R-mediated decrease in the phosphorylation level of mTOR. However, elevation of [Ca^2+^]i by ionomycin significantly abolished the effects of PPF on the expression of these proteins, indicating the involvement of [Ca^2+^]i in the PPF-mediated regulation of CaMKKβ/AMPK/mTOR signaling. Of note, overexpression of CaMKKβ or activation of AMPK pathway by AICAR significantly abrogated the PPF-mediated decrease in CaMKKβ protein expression and p-AMPK/AMPK ratio plus the increase in p-mTOR/mTOR ratio (Fig. 6b and c).

Subsequently, we investigated the effects of activation of CaMKKβ/AMPK/mTOR pathway on the PPF-mediated suppression of autophagy. Data revealed that activation of CaMKKβ/AMPK/mTOR pathway by overexpression of CaMKKβ and AICAR treatment significantly abolished the PPF-mediated autophagy suppression, as evidenced by an increase in LC3-II and Beclin-1 protein expression plus GFP-LC3 puncta, and a decrease in p62 compared with the PPF alone-treated group (Fig. 6d-e).

Collectively, these results indicated that Ca^2+^/CaMKKβ/AMPK/mTOR signaling was involved in the PPF-mediated suppression of autophagy in OGD/R-exposed neurons.

## Discussion

PPF has been widely used in anesthesia induction, maintenance, and intensive care. In addition to its sedative and hypnotic effects, PPF has also been shown to exert neuroprotective effects on cerebral I/R injury (Li et al. 2013; Wang et al. 2016). Of note, PPF could reduce both cerebral blood flow and cerebral metabolic rate of oxygen, whereas could not cause the risk of increasing intracranial pressure compared with other anesthetic drugs. A previous study demonstrated that PPF (1–50 μM) which was added during OGD/R period exerted a neuroprotective effect against OGD/R-induced cell injury in primary hippocampal neurons (a model of cerebral I/R in vitro), whereas PPF (100–200 μM) caused a more severe injury compared with OGD/R group (Wang et al. 2016). In accordance with this, our study also shows that PPF treatment (0.03 and 0.05 mM) during OGD/R period significantly antagonized OGD/R-triggered cell injury in primary rat cerebral cortical neurons (a model of cerebral I/R in vitro).

Recent studies concerning the neuroprotective mechanisms of PPF have focused on autophagy (Cui et al. 2012; Noh et al. 2010). Blood reperfusion following cerebral ischemia often leads to inflammation, oxidative stress, and endoplasmic reticulum stress, which can induce autophagy (T Y, 2013). OGD/R exposure is generally used to induce cerebral I/R injury and has been shown to induce autophagy in cultured cortical neurons (Qin et al. 2015). Basal autophagy plays an important role in the removal of excess proteins in neurons, but abnormal basal autophagy is related to neurodegenerative diseases (Tammineni & Cai, 2017). In ischemic brain injury, autophagy is a double-edged sword and has controversial functions (Peng et al. 2018; Hua et al. 2018; Zheng et al. 2018). It has been shown that increased autophagy acts as a compensatory protective mechanism for I/R injury (Su et al. 2014). For example, (Peng et al. 2018) showed that mitofusin 2, a mitochondrial fusion protein, could ameliorate cerebral I/R injury mainly through promoting autophagy in primary cultured neurons. In contrast, (Zheng et al. 2018) proposed that Bilobalide, an active component of *Ginkgo biloba* extract, inhibited autophagy and promoted angiogenesis following focal cerebral I/R, and thereby may be a potential agent for improving self-repair after ischemic stroke. Furthermore, (Wen et al. 2008) suggested that inhibition of autophagy by 3-methyladenine (3-MA) and bafliomycin A1 (BFA) reduced infarct volume, brain edema, and motor deficits in a rat model of permanent focal cerebral ischemia. (Li et al. 2017) also stated that inhibition of autophagy played a beneficial role in modulating neurological deficits after I/R observed under conditions of a lower level of estradiol.

Anesthetic agents have been shown to modulate autophagy in cardiac, neuronal, and other cell types. Autophagy is a process that must be carefully regulated as too much or too little autophagy may lead to cellular death. In some cases, upregulated autophagy was beneficial, whereas in others the opposite is true. The vast majority of studies demonstrate that anesthetic modulation of autophagy is beneficial for cell survival (Ye & Zuo, 2017). For example, (Zhou et al. 2016) showed that sevoflurane treatment activates autophagy, which antagonizes sevoflurane-induced apoptosis in H4 human neuroglioma cells. (Kwon et al. 2015) demonstrated that remifentanil protects human keratinocytes against hypoxia-reoxygenation injury through activation of autophagy. (Rao et al. 2017) proposed that isoflurane preconditioning alleviated murine liver I/R injury by restoring AMPK/mTOR-mediated autophagy. However, the findings of this study revealed that PPF antagonized OGD/R-triggered neuronal injury, which might be mediated, at least in part, via inhibition of autophagy. Our results were in agreement with the study, which showed that PPF prevented cerebral ischemia-triggered autophagy activation and cell death in the rat hippocampus after a cerebral I/R insult (Cui et al. 2013). Another study, which demonstrated that PPF exerted protective effects on neuronal PC12 cells after OGD injury via inhibition of autophagy, also confirmed the beneficial role of autophagy inhibition in cerebral I/R injury (Cui et al. 2012). However, unlike the protection of autophagy inhibition by PPF described above, a more recent study has been shown that PPF postconditioning protects H9c2 cardiac myoblast cells from hypoxia/reoxygenation injury by inducing autophagy (Li et al. 2018). The reasons for this discrepancy in the role of autophagy modulation by anesthetics are unclear but could relate to the degree to which autophagy is upregulated, or during when the autophagy cycle regulation occurs. Moreover, the controversial role of PPT in modulating autophagy may be attributed to the difference in cell type and setting, and ways of drug treatment.

Intracellular Ca^2+^ overload has been shown to be closely associated with I/R-induced apoptosis (Halestrap, 2006; Ren et al. 2017). (Wang et al. 2016) have stated that PPF could effectively reduce OGD/R-induced neuronal death in rat primary hippocampal neurons at 20 h after the injury for partially reducing calcium-overload during I/R injury. In this study, we found that PPF treatment significantly antagonized OGD/R-triggered [Ca^2+^]i elevation. Furthermore, [Ca^2+^]i elevation by the Ca^2+^ ionophore ionomycin significantly reversed the PPF-mediated amelioration of OGD/R-triggered cell injury. Recent studies have emphasized the correlation between Ca^2+^ and autophagy. It has been reported that an increase in [Ca^2+^]i activates CaMKKβ/AMPK signaling cascade, leading to the inhibition of mTOR signaling, and thus induction of autophagy (Shi et al. 2018; Zhang et al. 2015; Lee et al. 2012). (Hoyer-Hansen & Jaattela, 2007) have stated that an increase in free cytosolic Ca^2+^ induces autophagy via the CaMKKβ/AMPK/TSC1/2 (tuberous sclerosis complex 1 and 2)-Rheb-mTORC1 (mammalian target of rapamycin complex 1) signaling pathway. (Shi et al. 2018) have demonstrated that Saikosaponin-d, a sarcoplasmic/endoplasmic reticulum Ca^2+^ ATPase pump (SERCA) inhibitor, inhibits proliferation by up-regulating autophagy via the CaMKKβ/AMPK/mTOR pathway in ADPKD (autosomal dominant polycystic kidney disease) cells. (Zhang et al. 2015) reported that up-regulation of TRPC4 increased [Ca^2+^]i, which, in turn, activated the Ca^2+^/CaMKKβ/AMPK pathway, leading to mTOR inhibition and autophagy induction in vascular endothelial cells. Consistent with this, our mechanistic studies revealed that [Ca^2+^]i elevation by ionomycin significantly abrogated the PPF-mediated autophagy suppression in OGD/R-exposed neurons. Furthermore, ionomycin significantly abolished the PPF-mediated inhibition of CaMKKβ/AMPK/mTOR signaling. Additionally, activation of CaMKKβ/AMPK/mTOR signaling abrogated the PPF-mediated autophagy suppression.

## Conclusion

In summary, our findings demonstrate that PPF antagonized OGD/R-triggered neuronal injury, which might be mediated, at least in part, via inhibition of autophagy through Ca^2+^/CaMKKβ/AMPK/mTOR pathway. Our study provides novel insight into the mechanism underlying the neuroprotection of PPF in cerebral I/R injury.

## Additional file

Additional file 1: **Figure S1.** Block of [Ca^2+^]i enhanced the PPF-mediated amelioration of OGD/R-triggered cell injury. OGD/R-exposed neurons were pre-treated with an intracellular calcium chelator (BAPTA, 5 μM), followed by propofol (PPF, 30 μM in 0.1% DMSO) treatment. (A) (A) LDH release and (B) CCK-8 cell viability were performed to evaluate whether [Ca^2+^]i was involved in the PPF-mediated amelioration of OGD/R-triggered cell injury. Values are represented as the mean ± SD from three independent experiments. ^*^p < 0.05 vs. Control group; ^#^*p* < 0.05 vs. DMSO group; ^$^*p* < 0.05 vs. PPF group.(TIF 296 kb)

## Abbreviations

3-MA: 3-methyladenine
ANOVA: One-way analysis of variance
BFA: bafliomycin A1
CCK-8: Cell Counting Kit-8
DMEM: Dulbecco’s Modified Eagle’s Medium
FBS: fetal bovine serum
FITC: fluorescein isothiocyanate
GFP: Green fluorescent protein
HRP: horseradish peroxidase
I/R: ischemia-reperfusion
NSE: neuron-specific enolase
OGD: Oxygen-glucose deprivation
OGD/R: oxygen-glucose deprivation and re-oxygenation
PBS: phosphate-buffered saline
PI: propidium iodide
PPF: propofol
SD: standard deviation
