# Supplementary figures and images for: Propofol inhibited autophagy through Ca2+/CaMKKβ/AMPK/mTOR pathway in OGD/R-induced neuron injury
Source: Mol Med. 2018 Nov 23;24:58. doi: 10.1186/s10020-018-0054-1 (PMC6251140; doi:10.1186/s10020-018-0054-1)

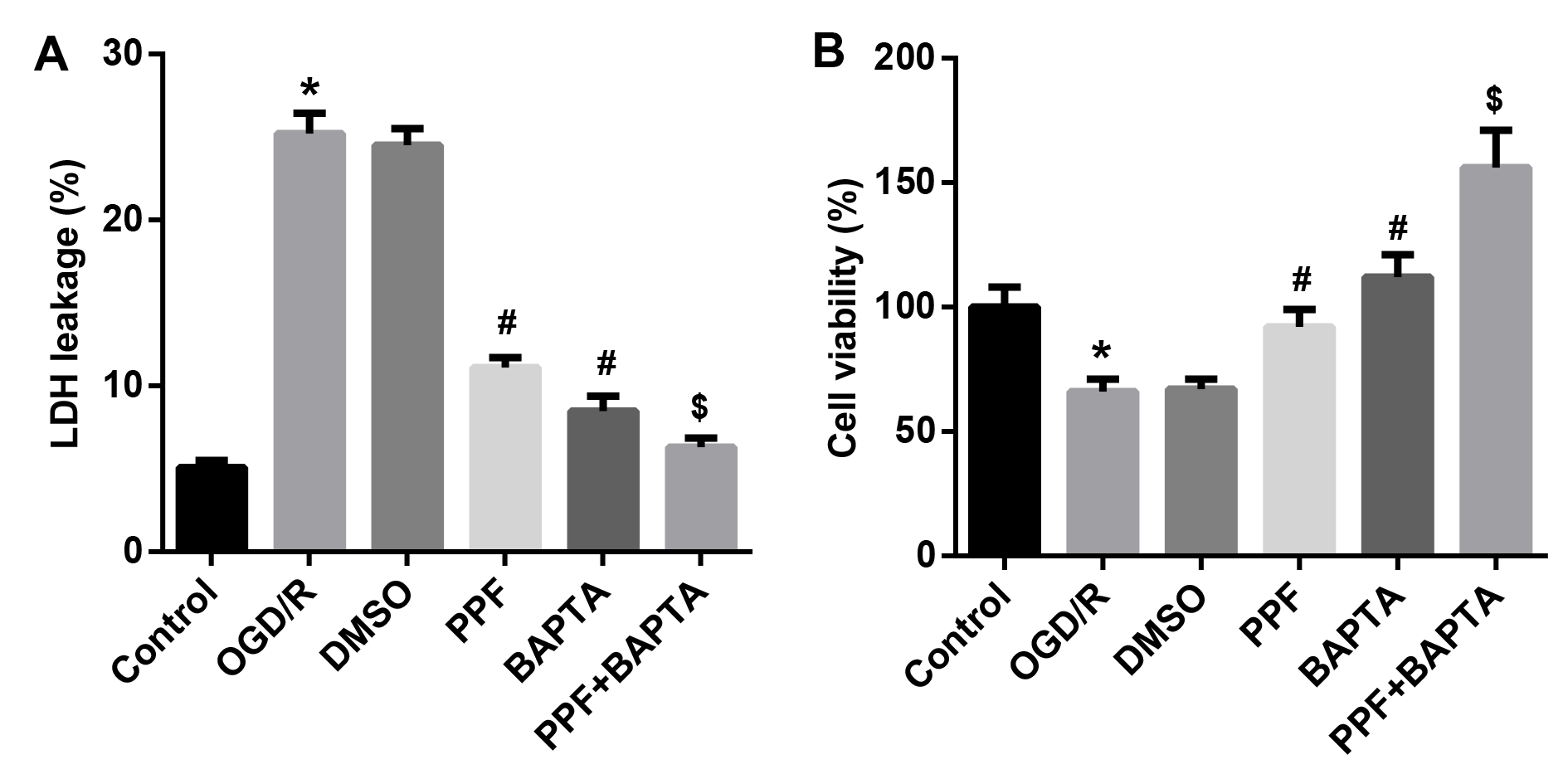

Supplement: Supplementary file 1 — Figure S1. Block of [Ca2+]i enhanced the PPF-mediated amelioration of OGD/R-triggered cell injury. OGD/R-exposed neurons were pre-treated with an intracellular calcium chelator (BAPTA, 5 μM), followed by propofol (PPF, 30 μM in 0.1% DMSO) treatment. (A) (A) LDH release and (B) CCK-8 cell viability were performed to evaluate whether [Ca2+]i was involved in the PPF-mediated amelioration of OGD/R-triggered cell injury. Values are represented as the mean ± SD from three independent experiments. *p < 0.05 vs. Control group; #p < 0.05 vs. DMSO group; $p < 0.05 vs. PPF group.(TIF 296 kb) [file 10020_2018_54_MOESM1_ESM.tif]
